# Spatial attention modulates time perception on the human torso

**DOI:** 10.3758/s13414-025-03025-6

**Published:** 2025-02-19

**Authors:** Bora Celebi, Müge Cavdan, Knut Drewing

**Affiliations:** Experimental Psychology - HapLab, Justus Liebig University Gießen, Otto-Behaghel-Str. 10F, 35394 Gießen, Germany

**Keywords:** Temporal processing, Attention, Haptics

## Abstract

Time perception is a fundamental aspect of human life, and is influenced and regulated by cognitive and sensory processes. For instance, spatial attention is found to modulate temporal judgments when resources are allocated to a specific stimulus location in vision and audition. However, it is unclear to what extent the attentional effects observed in vision and audition can be generalized to the tactile modality. Here, we study the effects of attentional cues on the time perception of tactile stimuli presented on the human torso. Across four experiments, we examined (1) the impact of visual versus tactile spatial cues, (2) the modulation of time perception by dynamic versus static tactile cues, (3) the role of spatial congruency between cue and target locations (front vs. back of the torso), and (4) the influence of cue-target intervals. Participants performed temporal bisection tasks, judging whether the vibrations following the cues were closer to short or long anchor durations. Tactile cues expanded the perceived duration of subsequent stimuli, with dynamic cues having a greater effect than static ones. While no congruency effects were observed for left and right torso locations, front-back congruency enhanced time expansion. The attentional effect peaked at a 100-ms cue-target interval. We conclude that the time-expanding effects of spatial attention extend to tactile stimuli on the human torso given that time expansion follows principles known from spatial attention.

**Supplementary Information:**

The online version contains supplementary material available at 10.3758/s13414-025-03025-6.

## Introduction

Estimating the duration of perceptual events is central to human behavior. Time perception is fundamental in constructing percepts of the physical world, but it does not have a dedicated sensory organ (Grondin, 2001). Temporal judgments are internal abstractions of perceived events; they co-occur with the perception of other dimensions that are related to their specific sensory processing systems (Allan, 1979). For example, when a moving object (e.g., a car) approaches, we estimate the time of arrival with visual and auditory cues to plan our actions. Along with its coherence with sensory processing of other stimulus characteristics, human time perception is affected by changes and manipulations applied to these (Eagleman, 2008). That is, judgments regarding the timing of events, such as subjective duration, temporal order, or simultaneity judgments are prone to disturbances regarding the perceptual properties of the stimulus and the cognitive functions related to the processing of the associated events (Arstila et al., 2019). Attention is one of the most prominent cognitive processes affecting temporal processing (Matthews & Meck, 2016). In particular, it has been shown that visual and auditory brief spatial cues expand the perceived time of a subsequent stimulus at the cued location. In order to investigate the generalizability of this effect, here we examine how spatial attention modulates temporal judgments within the tactile modality and on a rarely investigated somatosensory area: the human torso.

Attentional processes involve selecting objects, stimuli, locations, or events by directing limited perceptual resources to them. Through this, the processing of the specific sensory inputs that link to the selected items is improved, and the information processing is accelerated (Carrasco, 2011; Mulckhuyse & Theeuwes, 2010). Directing spatial attention can be achieved by endogenous or exogenous cues. The endogenous cues specifically transmit information about the features of the stimulus (e.g., its location) and the attention can be directed voluntarily towards the cued aspect (Posner, 1980). Exogenous cues direct the attention of the observer involuntarily and automatically by salient sensory information (Egeth & Yantis, 1997). When the observer’s attention is directed to the spatial location of a consecutive target stimulus, sensory processing of the target is facilitated. In typical visual experiments, the endogenous cue is an arrow pointing to a specific location (e.g., left vs. right), and the exogenous cue is a short salient visual stimulus appearing at a specific location. A target stimulus that has different features to the cue (e.g., a dot or a rectangle) is then delivered to the cued or the uncued location, and participants are asked to locate it. Reaction time is improved when the target appears at the cued location compared to the uncued location (Itti & Koch, 2000; Mulckhuyse & Theeuwes, 2010). Both endogenous and exogenous direction of attention result in performance gains in detection and discrimination along with increased perceptual magnitude and salience of the attended stimuli (Chun et al., 2010). Similar cue effects were also observed in the auditory (Kong et al., 2014; Mondor & Zatorre, 1995) and tactile (Bauer et al., 2006; Spence & Gallace, 2007; Spence & McGlone, 2001) modalities. For example Jones and Forster (2013) investigated endogenous and exogenous tactile cues in a spatial discrimination task. They used bimanual flutterlike versus continuous tapping stimulation as endogenous cues and informed participants about the side (left vs. right) to which each cue type refers. For exogenous cues, they used a brief unimanual spatially uninformative tap. Both congruent endogenous and exogenous cues decreased reaction times to subsequent tactile targets compared to incongruent cues.

Spatial attention can be shifted not only intramodally but also crossmodally. For instance, Spence and Driver (1997) demonstrated that auditory exogenous cues affect the localization performance of visual targets. Brief, non-informative auditory cues presented from either side of the room reduced the reaction times for subsequent congruent targets compared to incongruent targets, both auditory and visual ones. Gray and colleagues (2009) investigated the effects of tactile and auditory spatial cues on visual targets by presenting these cues and targets from different locations in front of a participant. Cues and target stimuli were presented from an array of seven locations in front of the participant, and tactile cues were presented to the forearm. Both tactile and auditory spatial cues facilitated visual localization, and with an increasing spatial distance between the cue and the target, localization reaction times increased. Ho and colleagues (2005, 2006) observed attentional effects of endogenous tactile cues from the torso (front vs. back) on visual speeded responses in a driving environment. In turn, spatial-visual and auditory cues have also been demonstrated to affect processing in the tactile modality (Spence et al., 1998). In general, the studies show that the presentation of spatial cues in one modality can facilitate the processing of a target stimulus in another modality at a corresponding spatial location (Driver & Spence, 1998; Hillyard et al., 2016; Ho et al., 2005; Spence & Driver, 1997; Spence & Soto-Faraco, 2020).

Note that moving cues have been found to direct spatial attention in visual, auditory, and tactile modalities particularly effectively (Glatz & Chuang, 2019; Hillstrom & Yantis, 1994; Ho et al., 2014). Ho and colleagues (2014) presented moving tactile cues at the waist to direct the participant's attention in a driving scenario. They used upward and downward-moving tactile cues, and measured braking response times to a visual target object approaching the participant. Regardless of motion direction and spatial configuration, moving cues decreased reaction times compared to static cues. These results suggest that dynamic tactile cues are more effective in directing spatial attention than static ones.

In the time-perception literature, endogenous and exogenous cue paradigms were employed to investigate the effect of spatial attention on temporal judgments (Block & Gruber, 2014). Mattes and Ulrich (Mattes & Ulrich, 1998) presented endogenous cues in the form of arrows that indicate the location of an upcoming stimulus. The judged duration of a subsequently presented black dot (duration: 70–123 ms) was longer when the stimulus was presented on the cued side indicated by the arrow, as compared to the uncued side. The authors concluded that visual spatial attention prolongs the perceived duration of brief events. Enns and colleagues (1999) reported that the temporal effect of endogenous visual cueing was larger with smaller stimulus onset asynchronies of 100, 200, and 500 ms, and it diminished with the highest, 1,600 ms. A similar dilation of subjective duration was observed in exogenous spatial cueing paradigms. In Yeshurun and Maron’s (2008) study, one of two possible visual target locations was cued by a bar just above that location in the cueing conditions. The exogenous spatial cues expanded the subjective time of a subsequent brief visual target as compared to spatially neutral cueing at the center of the screen. The findings were expanded with the comprehensive contribution of Seifried and Ulrich (2011). In a series of experiments involving various cue-target combinations, they found that the expansion effects of exogenous cueing on subjective durations can occur across a wide range of possible combinations involving visual cues, visual targets, and locations. They investigated stimulus durations ranging from 60 to 300 ms.

The effects of attentional processes and especially spatial cues on time perception have been studied in visual and auditory senses (e.g., Mattes & Ulrich, 1998). Research has shown that spatial cues expand the perceived duration of the targets in the same modality. To our knowledge, the link between attention and time perception mechanisms in the tactile modality, specifically in relation to the human torso, has not been previously explored. The human torso encompasses a large receptive area with different sensitivities where tactile sensory inputs are received (Lederman & Klatzky, 2009). In this study, we sought to determine the extent to which the impact of spatial attention on temporal judgments can be generalized, specifically to the tactile modality and the human torso. In four experiments, we investigated (1) how tactile and visual-spatial cues affect human tactile time perception on the torso compared to no cue, (2) whether dynamic tactile cues would be more effective at changing human subjective time perception compared to static cues (cf. Ho et al., 2014), (3) if spatially congruent tactile cues on the front versus back of the torso would expand duration judgments as compared to spatially incongruent cues, and (4) how the cue-target interval influences duration judgments. In all experiments, we first familiarized participants with short and long anchor durations of tactile stimuli on the torso. In the testing phase, we presented visual, static tactile, or dynamic tactile cues which were followed by target tactile stimuli in different durations. Participants judged the duration of these stimuli to be closer to the previously trained short or long anchor durations.

## Experiment 1

### Methods

#### Participants

An a priori power analysis was computed using G*Power ((Faul et al., 2007); power 80%, medium effect size of *partial η*^*2*^ = 0.06 for repeated-measures ANOVA, *α* = 0.05) which revealed a target sample size of 27. Twenty-six participants (15 females, age range: 20–27 years, *M* = 24.5, *SE* = 1.8 years) were recruited through a circular email at Justus-Liebig University, Giessen. None of the participants reported any somatosensory impairments and all reported normal or corrected-to-normal vision. Prior to the experiment, they provided written informed consent and were compensated 8€/h for their participation. Experimental procedures were approved by the local ethics committee of Justus-Liebig University, Giessen, LEK FB 06, in accordance with the Declaration of Helsinki without preregistration (Medical Association, 2013).

#### Apparatus, setup, and stimuli

A commercially available tactile vest (bHaptics TactSuit X40) was used to deliver tactile stimuli. The vest delivers approximately ~ 90 Hz vibrotactile feedback through each one of the 40 eccentric rotating mass (ERM) actuators. The vest was adjusted to an individual’s body size using the straps located on the sides. Participants stood in front of a height-adjustable table and their head positions were fixed by a chin rest. A screen (DELL, 27 in., 60 Hz) on the table was used to present the visual stimuli. The distance between the participants’ eyes and the center of the screen was approximately 60 cm. Noise-canceling headphones (Sennheiser Momentum 3) were used to present white noise at 70 dB which masked any noises caused by the actuators. The experiment was programmed in MATLAB 2021b using Psychtoolbox version 3.0.18 (Kleiner et al., 2007). Control commands were transmitted to the vest through Bluetooth using a custom-made Python script. A keyboard collected the participants’ responses.

**Fig. 1:**
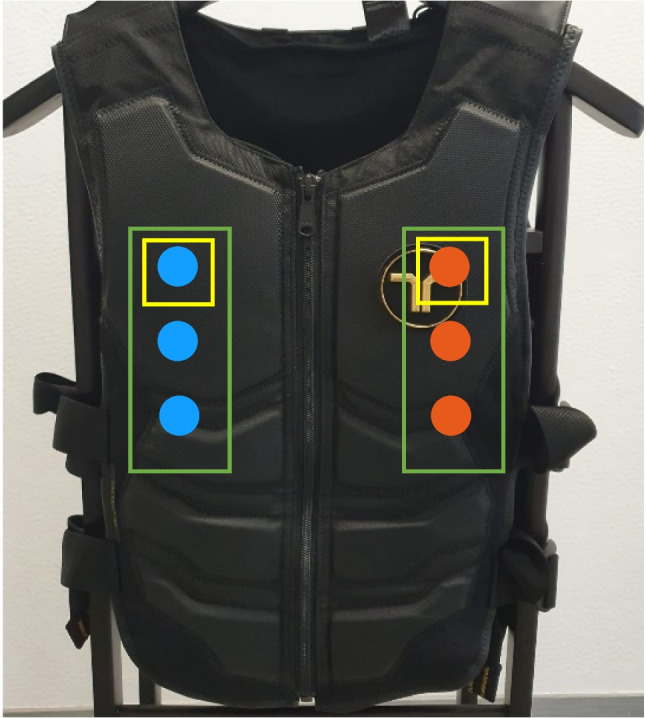
Vibrotactile vest and the actuator locations used in the experiment. Green rectangles depict the actuators of the tactile target stimulus and yellow rectangles depict the actuators of the tactile cue

Tactile target stimuli consisted of the simultaneous vibration of three different actuators on the vest, spanning from the upper chest to the mid-abdominal area on the front of the torso (Fig. 1, green rectangles). The vertical distance between two neighboring actuators was 12 cm, and the actuators were located 12 cm to the right or left side of the midline of the torso (Fig. 1). The vibration amplitude was 14.5 m/s^2^ root-mean-squared (RMS). This three-actuator target stimulus was presented to either the left or the right side of the midline of the torso (Fig. 1, green rectangles). In a temporal bisection task, the following stimulus durations were used: 300 (short anchor), 400, 500, 600, 700, and 800 ms (long anchor).

There were three different cue conditions: tactile, visual, and no cue. The tactile spatial cue was a 100-ms vibration delivered from the uppermost actuator from either the left or the right side (Fig. 1, yellow rectangles). The visual cue was a white flickering circle with a radius of 0.55°, and the center of the circle was 10° to the right or left of the screen center. Weber's contrast ratio between the cue and the background was 3.37. The circle was presented for every second frame, creating a flickering pattern for 100 ms. The cues were congruent with the tactile target stimulus, meaning both cue and target were presented from the same spatial location (right or left to torso midline). In the no-cue condition, there was no stimulus but a blank period of 100 ms.

#### Design and procedure

A within-participants design with the effect of cue (three levels: no, visual, and tactile cue) on time perception was used. In a temporal bisection task, participants judged the target durations. The task consisted of learning and test phases. In the learning phase, participants learned to distinguish the short (300 ms) and long (800 ms) target anchor durations. In the test phase, they indicated whether the target duration ranging from 300–800 ms (100-ms increments) was long or short.

Learning phase. First, participants trained with the anchor durations, followed by a practice. Tactile target stimuli were presented to the right or left side of the front torso for the anchor durations of 300 ms and 800 ms. Each stimulus was repeated five times in a randomized order for every combination (2 durations × 2 sides × 5 repetitions), culminating in a total of 20 trials. First, a tactile stimulus was presented, followed by a 500-ms blank period, and then by feedback on the center of the screen indicating whether the duration had been short or long (for 1,000 ms, Fig. 2a). After a 750-ms intertrial interval, the next stimulus was presented. A fixation cross was always present in the middle of the screen besides the feedback. Participants were instructed to memorize the duration of the stimuli. After this training phase, the same 20 target stimuli were presented in a randomized order as before. In each trial, following a 500-ms blank interval, participants categorized the duration of each stimulus as “short” or “long” using the “left arrow” and “right arrow” keys. After the response, feedback on whether the response had been correct or not was presented for 1,000 ms (Fig. 2b). The next trial started 750 ms after the end of the feedback window. The average accuracy for the practice phase was 0.96.

**Fig. 2:**

Time course of a trial in the (**a**) training phase and (**b**) practice phase of the learning

**Fig. 3:**
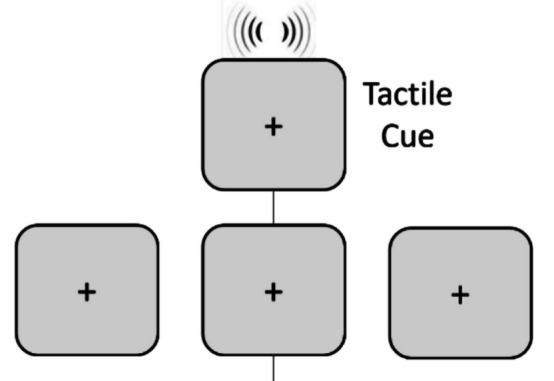
An example trial of each cue condition (i.e., tactile, no, and visual cue from top to bottom). If the target is in one of the anchor durations, feedback about the validity of their responses was presented for 1,000 ms after the response

Test phase. The test phase of the temporal bisection task consisted of three separate blocks, one per cue condition: tactile, visual, or no cue. Each trial started with a 750-ms fixation cross on the screen. In the visual cue condition, this was followed by a 100-ms visual cue to the right or the left side of the screen. In the tactile cue condition, a 100-ms vibration was presented left or right to the torso as a cue. In the no-cue condition, instead of a visual or a tactile cue, there was a 100-ms blank interval with a fixation cross (Fig. 3). In all conditions, 500 ms thereafter, a tactile stimulus ranging from 300 to 800 ms in 100-ms increments was delivered to the same side. The question “short or long?” appeared on the screen 100 ms after the offset of the tactile stimulus. The task was to categorize the duration of the tactile target as “short” or “long” using the left and right arrow keys from the keyboard. They had 3,000 ms to categorize the duration, otherwise the trial ended. If the target tactile stimulus lasted for one of the anchor durations (300 or 800 ms), participants obtained feedback on the correctness of their response through a text on the screen that prompted”Correct” or”Wrong” for 1,000 ms. If the target duration was not one of the anchors, no feedback was provided. Then, the next trial began.

Block order was balanced using a Latin square design across participants. In each block, participants were presented with tactile target stimuli of the six durations at two locations (left or right torso), with 12 repetitions (i.e., 144 trials per block). The trial order in each block was randomized. The experiment lasted approximately 20 min.

### Data analysis

We fitted logistic psychometric functions to the proportion of “long” responses using the Psignifit 4 toolbox in MATLAB 2021b (Schütt et al., 2016) per condition and participant. This toolbox estimates the function parameters using Bayesian inference. Higher and lower asymptote parameters (gamma and lambda, *M* = 0.011, *SE* = 0.021) were assumed to be equal while alpha and beta parameters were left free. Bisection points (BPs) were calculated from the psychometric function as the stimulus duration that had a 50% frequency of “long” responses. The Just Noticeable Difference (JND) was defined by half of the difference between the stimulus durations with 25% and 75% frequencies of “long” responses. The Weber Fractions (WFs) were the ratio between JND and BP. We reported WFs as a measure of discrimination sensitivity throughout the results section because they indicate changes in the underlying timing mechanisms (Grondin, 2014). JND results are additionally reported in the Online Supplementary Materials. There was no significant difference of BPs between left and right sides of the torso revealed by a pairwise t-test, *t*(25) = 0.26, *p* = 0.76, therefore we aggregated the data from both sides.

## Results

**Fig. 4:**
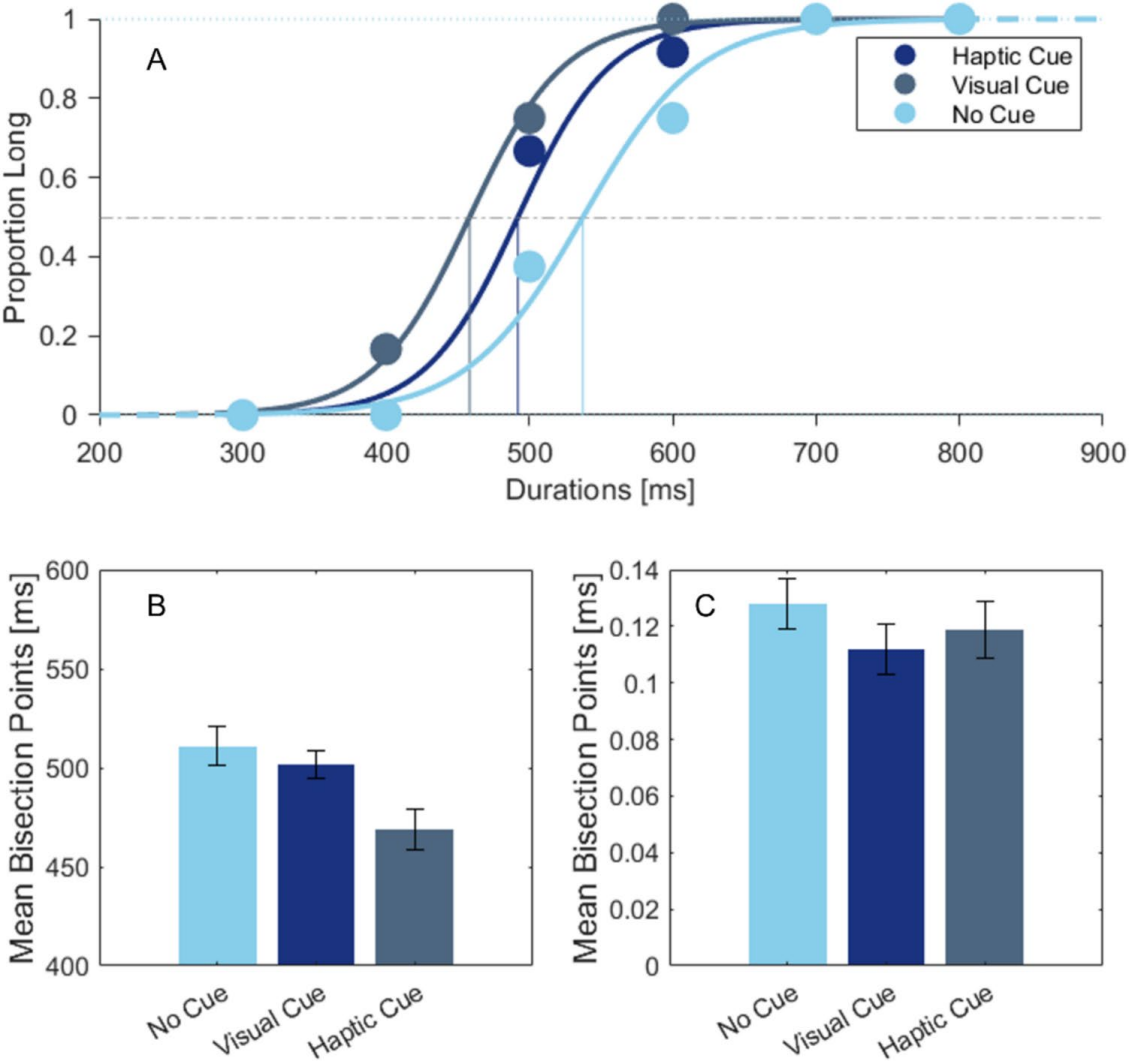
Psychometric functions of a single participant (**A**), mean bisection points (**B**), and mean Weber Fractions (**C**) in the first experiment. Error bars refer to the standard error of the mean

Individual BPs were submitted to a univariate repeated-measures analysis of variances (ANOVA) to test the effect of the cue (visual cue, tactile cue, no cue) on the perceived time of the tactile stimulus. Mauchly’s test did not indicate a significant violation of the sphericity assumption, *χ*^*2*^ (2) = 2.712, *p* = 0.258. The main effect of the cue condition was statistically significant, *F*(2, 50) = 10.769, *p* < 0.001, partial *η*^*2*^ = 0.301. In Bonferroni-adjusted pair-wise post hoc comparisons, the BP in the vibrotactile cue condition (*M* = 468.90, *SE* = 9.86) was significantly lower than in the no-cue condition (*M* = 511.28, *SE* = 10.05) at *p* < 0.001, and than in the visual cue condition (*M* = 502.18, *SE* = 7.22) at *p* = 0.017 (Fig. 4A). These results reveal that after the tactile cue, participants estimated the duration of the stimuli to be longer than after the visual and no-cue conditions. However, there was no significant difference between the no-cue and visual cue conditions, *p* = 0.997.

To test the possible sensitivity differences between cue conditions, another ANOVA was conducted on the WFs (Fig. 4B). Mauchly’s test did not indicate a significant violation of the sphericity assumption *χ2* (2) = 2.434, *p* = 0.296. The main effect of the cue on the WFs was statistically significant: *F(*2,50) = 3.252, *p* = 0.047, partial *η*^*2*^ = 0.115. Bonferroni-adjusted post hoc comparisons showed significantly higher WFs in the tactile (*M* = 0.128, *SE* = 0.009) than in the visual cue condition (*M* = 0.112, *SE* = 0.009) at *p* = 0.020, indicating that duration detection sensitivity was slightly worse in the tactile than in the visual cue condition. However, there was no significant difference between no-cue (*M* = 0.119, *SE* = 0.010) and visual cue (*p* = 0.883) and between no-cue and tactile cue conditions (*p* = 0.609). Results regarding JNDs can be found in the Online Supplementary Material in Tables 1 and 2.

## Experiment 2

The first experiment showed that tactile cue expands the perceived time of the following tactile target. We posit that this effect can be attributed to tactile spatial attention. To further test this idea, in the second experiment, we examined the impact of two additional variables known to affect spatial attention on time perception. Specifically, we investigated the influence of dynamic tactile cues on time perception, since they have been shown to attract tactile attention more effectively than static cues (Ho et al., 2014). Also, while all cue-target locations were congruent in Experiment 1, we examined how spatial congruency between the tactile cue and the target would impact subjective timing judgments in Experiment 2, since previous research has demonstrated that congruency strongly influences attention.

### Methods

#### Participants

Because the results of the first experiment yielded large effect sizes, a priori power analysis was computed now assuming medium to large effect size (power 80%, partial *η*^*2*^ = 0.08 for repeated-measures ANOVA, α = 0.05) that revealed a sample size of 21. Twenty-four participants aged between 20 and 30 years (*M* = 24.08, *SE* = 3.03 years) were recruited through a circular email from Justus-Liebig University, Giessen. Participants did not report any somatosensory impairments and reported normal or corrected-to-normal vision. They provided written informed consent prior to the experiments and were compensated 8€/h for their time.

#### Stimuli and apparatus

We used the same apparatus and similar tactile target stimuli as in Experiment 1. In contrast to Experiment 1, tactile target stimuli were presented to either the front or back of the vest, with amplitudes of 12.9 (only front) or 14.5 m/s^2^ (front or back). The two front amplitudes were chosen to establish both a condition of *physical* equality to back amplitudes as well as a condition of *perceived* equality to back amplitudes (because front stimuli are perceived slightly more intense; Celebi et al., 2022). Participants wore a generic cotton t-shirt to eliminate the dampening of tactile stimulus caused by different kinds of clothing.

**Fig. 5:**
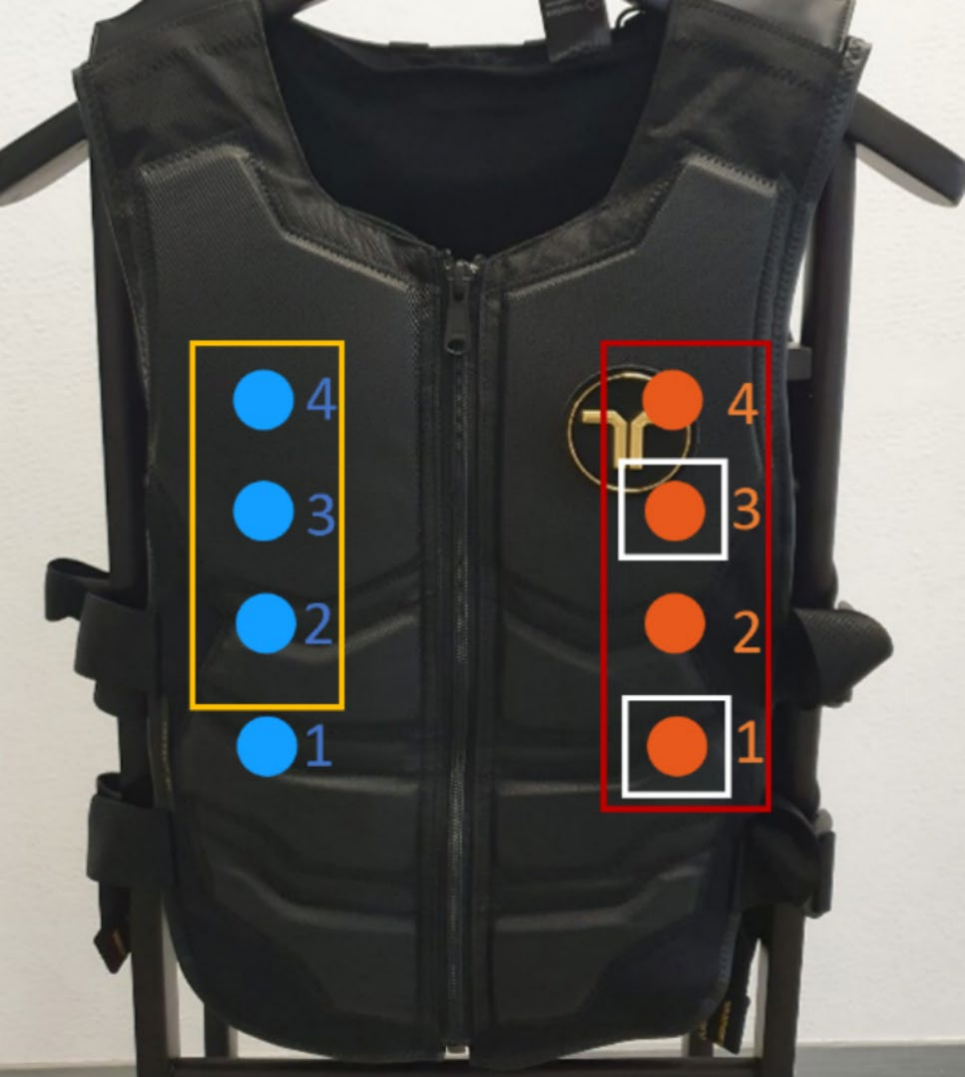
Actuator locations on the vibrotactile vest. The red rectangle depicts actuators used for the dynamic cue condition, white rectangles depict actuators used for the static cue condition, and the yellow rectangle depicts the actuators used for the target stimuli. The red numbers refer to the order of actuation in the dynamic cue. Locations of the actuators are approximate and both cues and targets can be delivered both on the right or the left (either back or front) locations

**Fig. 6:**
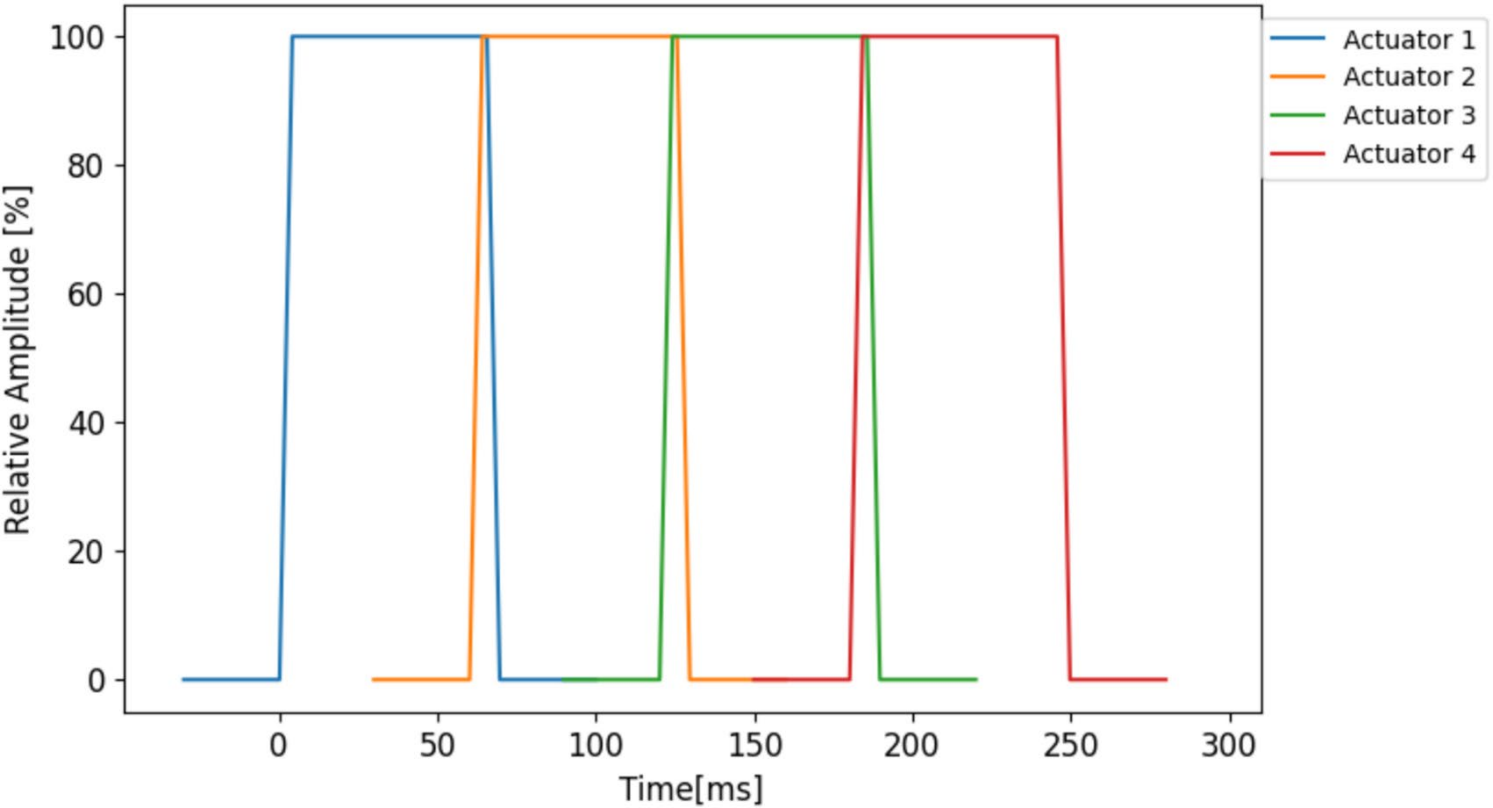
Illustration for the haptic motion illusion pattern used for the dynamic cue condition. The amplitude is shown as the percentage of the maximum amplitude (12.9 or 14.5 m/s.^2^ depending on the condition)

In Experiment 2, both dynamic and static cues were used. Static cues were presented simultaneously from actuators 1 and 3 (Fig. 5) from one side of the torso (left vs. right) for a fixed period of 250 ms. Dynamic cues utilized the apparent haptic motion illusion (Lakatos & Shepard, 1997) to induce the perception of moving stimuli. Four actuators (1, 2, 3, 4) were placed between the middle-lower abdomen and upper chest area and were actuated successively as follows (cf. Figure 5): Actuator 1 vibrated for 70 ms; 60 ms after actuator 1’s onset, actuator 2 started vibrating for 70 ms so that both actuate for 10 ms synchronously. Actuator 3 started vibrating 60 ms after actuator 2’s onset and actuator 4 started vibrating 60 ms after actuator 3’s onset. The total duration of the pattern was 250 ms; the amplitude of each actuator linearly increases in the first 10 ms and linearly decreases to zero in the last 10 ms, i.e., in the 10-ms period of overlap between actuators. The movement started from the lowest position (low-mid abdomen) on the torso and finished at the highest one (upper chest), cueing a direction towards the upper side of the body. Note that the total actuation time was lower for the dynamic cue (4 × 70 ms = 280 ms actuation) than for the static cue (2 × 250 ms = 500 ms). Cue and target stimuli were both presented on either the front or the back side of the torso. The left versus right side location of the cue and target was congruent in half of the trials and incongruent in the other half Fig. 6.

#### Design and procedure

A 2 (cue type: dynamic and static) × 2 (congruency: congruent and incongruent) × 3 (torso side: front, front-perceived intensity, and back) within-participants design was used. Cues and target stimuli were equally often presented on the left and the right side of the body, and cues and targets were equally often congruent or incongruent concerning their left–right location. We used two front conditions because a study had shown that vibration amplitude on the back needs to be approximately 12% higher than at the front to be perceived as equally intensive (Celebi et al., 2022). In one front condition, vibration amplitudes were physically equated with those on the back (14.5 m/s^2^), in the other they were reduced to be perceptually equal (12.9 m/s^2^). The participant’s task was to judge target duration in a temporal bisection task. As in Experiment 1, the anchor durations were 300 ms (short) and 800 ms (long), and target durations in the main experiment were 300, 400, 500, 600, 700, and 800 ms. The experiment lasted approximately 45 min.

The learning phase (average accuracy 0.95) was similar to Experiment 1; in addition, we used 40 randomly ordered trials per phase ([300 vs. 800 ms] × [front vs. back] × [left vs. right] × 5 repetitions, amplitude always 14.5 m/s^2^), and participants used the “U” and “N” keys to categorize durations in the practice phase. The main experiment consisted of three blocks, one per torso side condition; the order was balanced across participants following a Latin square design. Within each block, 336 trials were presented in random order, i.e., (6 durations) × (static vs. dynamic cue) × (left vs. right cue) × (congruent vs. incongruent) × (7 repetitions). A single trial in the main experiment followed the procedure in Experiment 1 except that the cue was presented for 250 ms, and the interval between cue and target was only 100 ms.

## Results

To assess the tactile cue effects on time perception, we conducted a 2 congruency (congruent and incongruent) × 2 cues (static and dynamic) × 3 torso side (front, front-reduced, and back) repeated measures ANOVA on individual BPs. The sphericity assumption was violated for the main effect of the torso side, *χ2* (2) = 16.039 at *p* < 0.01, and the corresponding results were corrected using Greenhouse–Geisser (Greenhouse & Geisser, 1959).

**Fig. 7:**
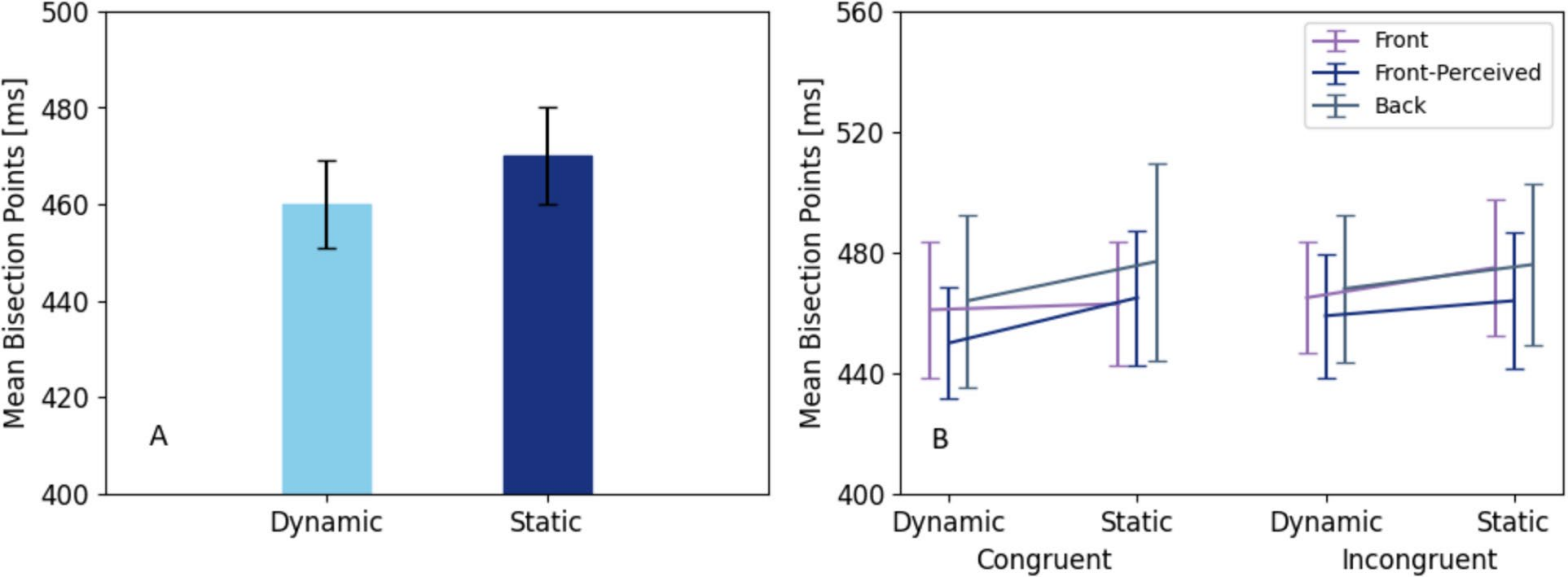
Mean bisection points as a function of cues (**A**) and as a function of all combinations of conditions (**B**). Error bars refer to the standard error of the mean

The main effect of tactile cue type on the BPs was statistically significant: *F*(1,23) = 7.954, *p* = 0.010, partial *η*^*2*^ = 0.257. BPs for the dynamic cues (*M* = 460.82, *SE* = 9.12) were significantly lower than for static cues (*M* = 472.24, *SE* = 9.18). This result reveals that a tactile stimulus was perceived longer after a dynamic cue as compared to after a static cue (Fig. 7).

Neither congruency nor torso side had an effect on the BPs, *F*(1,23) = 0.408, *p* = 0.529, partial *η*^*2*^ = 0.017, *F*(2,46) = 0.976, *p* = 0.355, partial *η2* = 0.041, respectively. Statistical analysis also did not reveal any significant interaction effects between cue type and congruency, *F(*1,23) = 0.902, *p* = 0.352, partial *η*^*2*^ = 0.038, torso side and cue type, *F*(2,46) < 0.019, *p* = 0.981, partial *η*^*2*^ = 0.002, torso side and congruency, *F*(2,46) = 0.072, *p* = 0.931, partial *η*^*2*^ = 0.003, or in the three-way interaction, *F*(2,46) = 0.095, *p* = 0.909, partial *η*^*2*^ = 0.004.

We also conducted a repeated-measures ANOVA on WFs (*mean* WF = 0.147) with the same design as the test on BPs. There was no significant main effect of cue type*: F*(1,23) = 0.003, *p* = 0.959, partial *η2* < 0.001, congruency, *F*(1,23) = 0.910, *p* = 0.35, partial *η*^*2*^ = 0.038, and torso side *F*(2,46) = 0.142, *p* = 0.868, partial *η*^*2*^ = 0.006. Also, interaction effects were not significant (all *p* < 0.778). Results of a repeated-measures ANOVA on JNDs are reported in Online Supplementary Material Table 3.

## Experiment 3

The second experiment did not reveal any congruency effects which is at odds with previous findings on how spatial cues drive attention (Seifried & Ulrich, 2011). We posit that this could be attributed to the spatial proximity of actuation locations on the torso (right, left) and the dissipating nature of the vibrotactile stimuli. As a result, the spots of spatial attention might not have been effectively different in the congruent and incongruent conditions of Experiment 2. Therefore, we conducted Experiment 3 in order to test if spatial cue would affect timing judgments on the torso, when the locations of the target and cues were distinct enough. We choose the target and cue locations as front or back because for these locations spatial attention effects have been previously demonstrated (Ho et al., 2005, 2006).

### Methods

#### Participants

Twenty-four participants, as in Experiment 2, were recruited through a circular email from Justus-Liebig University, Giessen (age range: 21–34 years; *M* = 24.25, *SE* = 2.75 years). Participants did not report any somatosensory impairments and reported normal or corrected-to-normal vision. They provided written informed consent prior to the experiments and were compensated 8€/h for their time.

#### Stimuli and apparatus

**Fig. 8:**
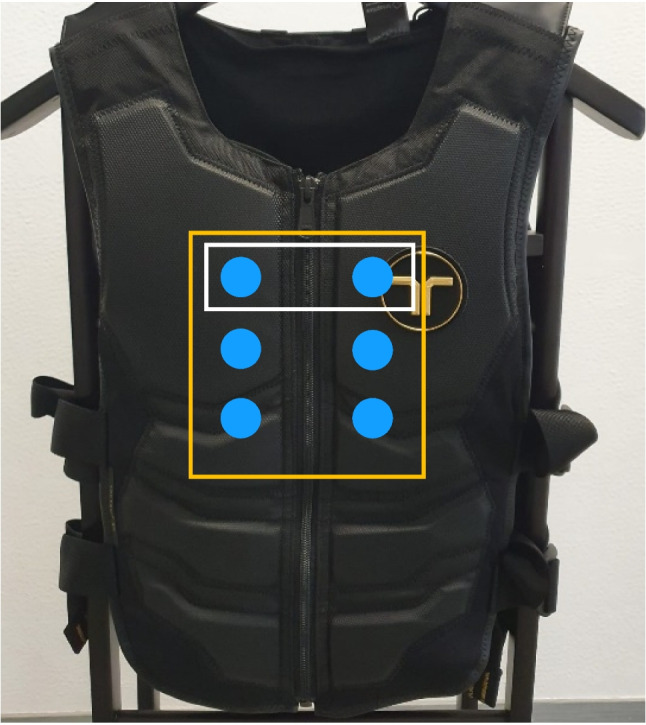
Actuator locations used in Experiment 3. Blue spheres illustrate actuators. Yellow rectangle depicts actuators used as the target and white rectangle depicts actuators used as the cue. Symmetrical actuators on the back of the vest were used for the back cue and targets

We used the same apparatus and similar tactile target stimuli as in Experiments 1 and 2. Tactile target stimuli were presented to either the front or back of the vest, with amplitudes of 12.9 (only front) or 14.5 m/s^2^ (back) in order to match the perceived intensity of stimulation between front and back (Celebi et al., 2022). Also, the target and cue were presented from the center of the vest instead of right and left sides. Target stimuli included the simultaneous presentation of six middle actuators of the vest (three vertical by two horizontal placement) spanning from the upper-chest to mid-abdominal area. Cue stimuli consisted of only the two upper-most actuators (Fig. 8).

#### Design and procedure

A within-participant design with a factor of congruency was used. Cues and targets were equally presented on the front and back side of the torso and were equally either congruent or incongruent. The participants judged the target duration in a temporal bisection task with the same durations as the previous experiments. The learning and practice phases (average accuracy 0.94) were similar to the previous experiments but we used 20 trials per phase (2 [300 vs. 800 ms] × 2 [front vs. back] × 5 repetitions). In the main experiment phase, there were 168 trials, i.e., 6 (durations) × 2 (front vs. back cue) × 2 (congruent vs. incongruent) × 7 (repetitions). A single trial followed the same procedures as in the second experiment.

## Results

**Fig. 9:**
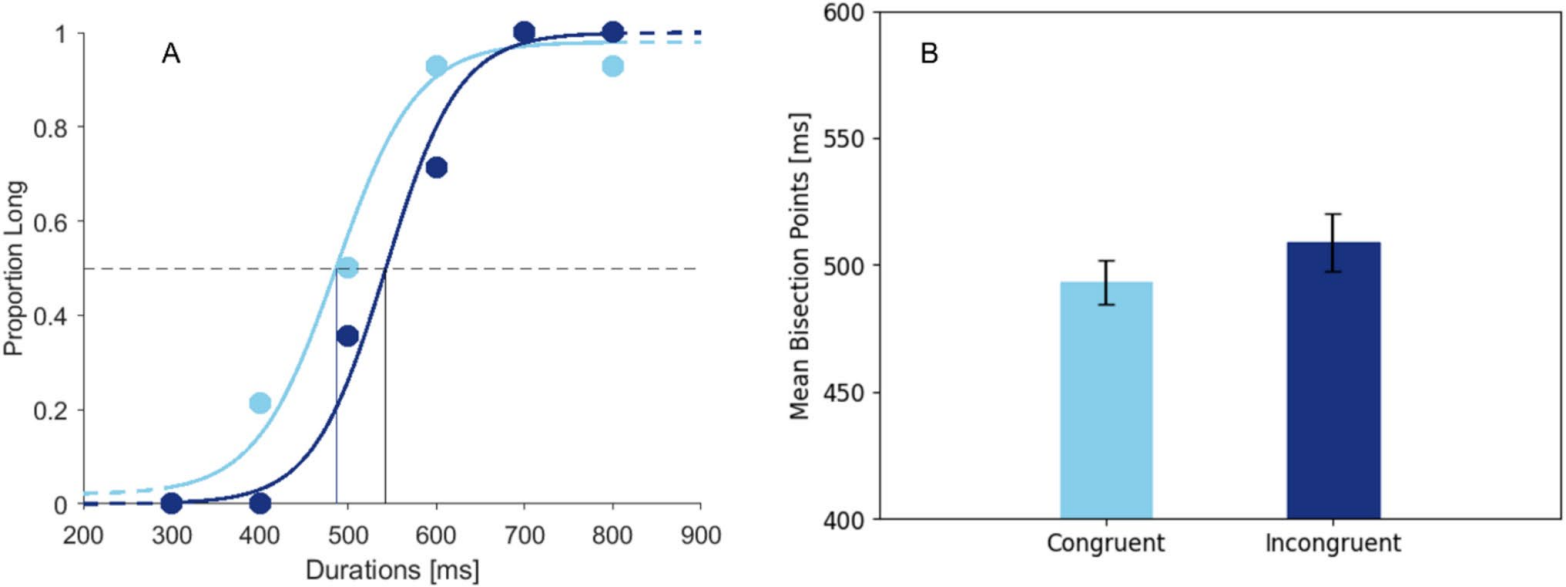
Psychometric function fits of a single participant (**A**) and mean bisection points as a function of congruency (**B**). (A) Dots refer to the individual proportion of long responses for each duration. The horizontal dashed line is at 50% long responses, the corresponding vertical lines depict the bisection points. (B) Error bars refer to the standard error of the mean

We ran a one-tailed paired-samples *t*-test on BPs between congruent and incongruent conditions. BPs in the congruent condition (*M* = 493.11, *SE* = 8.55) were significantly lower than BPs in the incongruent condition (*M* = 508.81, *SE* = 11.21) *t*(23) = 1.86, *p* < 0.038, *d* = 0.38, which shows that targets were perceived longer when they were congruent with the cues (Fig. 9). Furthermore, a two-tailed paired-samples *t*-test on WFs showed that there was no significant difference between congruency conditions, *t*(23) = 1.41, *p* = 0.170, *d* = 0.29 and *t*(23) = 1.69, *p* = 0.105, *d* = 0.34, respectively. This shows that time discrimination performance was similar across conditions. Results regarding JNDs are reported in Online Supplementary Material Table 4.

## Experiment 4

The third experiment showed that spatial congruency effects were present when the cue locations were sufficiently distinct. Findings from Experiment 3 further corroborate the hypothesis that attentional processes mediate the time expansion. In the fourth experiment, we varied the cue-target interval. If time expansion is indeed due to attention, the expansion effect should peak at a certain interval and be smaller for a long or very short interval corresponding to the temporal dynamics of spatial attention (Chica et al., 2014). We manipulated the interval between cue and the target in four steps: in one step the target was as close as possible to the cue (~ 5 ms) so that attentional processes cannot yet be expected. The remaining intervals were 100, 300, and 500 ms, and cues and target were either congruent or incongruent in terms of front versus back of the torso.

### Methods

#### Participants

Participants were recruited through a circular email from Justus-Liebig University, Giessen. Twenty-six participants, similar to Experiments 2 and 3, and aged between 21 and 35 years (*M* = 27.76, *SE* = 4.08 years) were included into the data set. Two further participants were discarded from the analysis since their responses in at least one condition did not reach the 50% point which rendered the BP estimates unreliable. Participants did not report any somatosensory impairments and reported normal or corrected-to-normal vision. They provided written informed consent prior to the experiments and were compensated 8€/h for their time.

#### Stimuli and apparatus

We used the same apparatus and similar tactile target stimuli as in Experiment 3. The only difference was the interval between cue and the target. The cue-target intervals were 5, 100, 300, and 500 ms.

#### Design and procedure

A within-participant design with the factors congruency and cue-target interval was used. The participants judged the target duration in a temporal bisection task with the same durations as the previous experiments. The learning and practice phases (average accuracy 0.95) were the same as in the third experiment. In the main experiment phase, there were 672 trials, i.e., 6 (durations) × 2 (front vs. back cue) × 2 (congruent vs. incongruent) × 4 (intervals) × 7 (repetitions). A single trial followed the same procedures as in the third experiment.

## Results

**Fig. 10:**
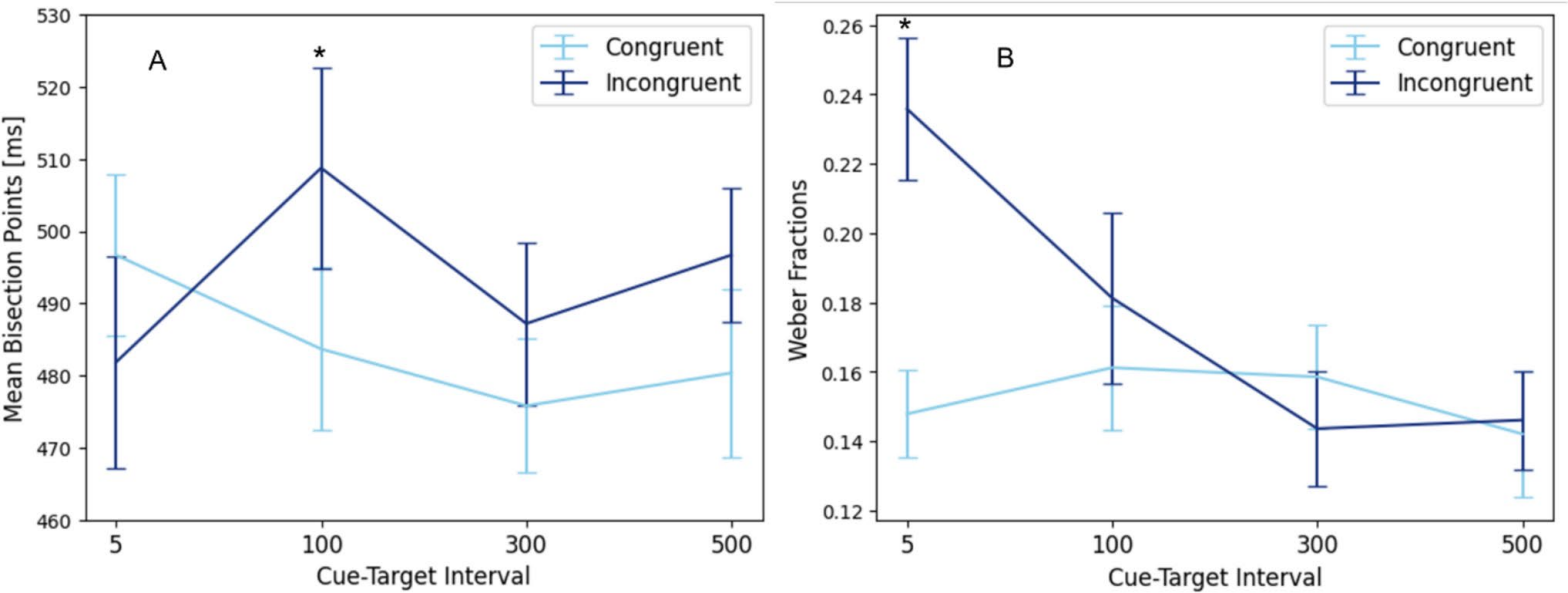
Mean bisection points (**A**) and mean Weber Fractions (**B**) as a function of congruency and cue-target interval. Error bars refer to the standard error of the mean. Asterisks correspond to p < 0.05

To assess the effects of congruency and cue-target interval we ran a 2 (congruent and incongruent) × 4 (interval) repeated-measures ANOVA on BPs. The sphericity assumption was violated for the main effect of cue-target interval, *χ2* (2) = 18.533 at *p* < 0.01, and the corresponding results were corrected using Greenhouse–Geisser. There was no significant difference between cue-target intervals, *F*(3,75) = 1.24, *p* > 0.29, partial *η*^*2*^ = 0.05. As expected, the main effect of congruency was significant *F*(1,25) = 3.96, *p* < 0.029 (one sided), partial *η*^*2*^ = 0.14. BPs in the congruent condition (*M* = 484.12, *SE* = 9.92) were smaller than in the incongruent one (*M* = 493.61, *SE* = 10.54). The interaction between interval and congruency was significant *F*(3,75) = 5.30, *p* < 0.004, partial *η*^*2*^ = 0.17. Bonferroni-corrected post hoc tests revealed that congruent BPs (*M* = 484.16, *SE* = 10.82) were significantly smaller than incongruent ones (*M* = 508.84, *SE* = 13.62) when the interval was 100 ms, *p* < 0.03 (Fig. 10).

In order to assess differences in sensitivity, we also submitted WFs to the repeated-measures ANOVA with the same design. The test revealed a significant main effect of congruency *F*(1,25) = 18.61, *p* < 0.001, partial *η*^*2*^ = 0.42, and a significant main effect of cue-target interval *F*(3,75) = 6.79, *p* < 0.004, partial *η*^*2*^ = 0.21. There was also a significant interaction effect *F*(3,75) = 7.91, *p* < 0.001, partial *η*^*2*^ = 0.24. Because the interaction was significant, we conducted post hoc comparisons by comparing congruent and incongruent conditions per each target-cue interval: When the cue-target interval was 5 ms, WFs in the incongruent condition (*M* = 0.147, *SE* = 0.012) were significantly higher than congruent condition (*M* = 0.236, *SE* = 0.021) at *p* < 0.001, hinting at differences in timing processes, but the other comparisons did not reach significance (Fig. 10). Results regarding JNDs are reported in Online Supplementary Material Tables 5 and 6.

## General discussion

In this study, we investigated the effect of attentional processes on human time perception on the torso. The first experiment showed that tactile cues prolong the perceived duration of the tactile stimuli. In the second experiment, we found that after the presentation of dynamic tactile cues, which are thought to be especially effective at directing attention, perceived time expanded more significantly compared to conditions where static cues were presented. The third experiment showed that spatially congruent tactile target and cue combinations expand the perceived duration of the target as compared to incongruent ones, which further corroborates a role for spatial attention. Finally, in line with the dynamics of attentional processes, the fourth experiment revealed that this effect peaks at a cue-target intervals of 100 ms. Based on these results, we conclude that attentional processes expand the perceived time for tactile stimuli on the torso. Unexpectedly, in Experiment 2, left–right spatial congruency between cue and target on the torso did not affect the timing judgments while front-back congruency did. This might indicate that spatial attention on the torso is not sufficiently focused to dissociate between the left and right sides. Overall, our findings indicate that expansion effect of spatial attention on time perception is a more general phenomenon than previously recognized, as it extends to the tactile modality on the human torso, with dynamic cues having a larger effect.

The tactile cues used in this study likely functioned as exogenous cues. In all experiments tactile cues were given at the potential locations of the targets. In Experiments 2, 3, and 4, the cues were non-predictive of the upcoming target location, and specifically, in Experiments 3 and 4, we still observed effects of congruent cues on time perception. These effects indicate involuntary attentional shifts driven by exogenous cues, which modulate perceived time independent of the cue’s predictive value (Egeth & Yantis, 1997).

In detail, Experiment 1 showed that the subsequent presentation of a tactile stimulus after a tactile cue at the location of the target felt longer than the presentation of an uncued tactile stimulus. This result is in agreement with the findings of Seifried and Ulrich (2011), who showed in an exhaustive series of experiments that different kinds of exogenous visual cues can expand the perceived time of a subsequent visual stimulus. Furthermore, it has been shown that spatial attention degrades the temporal sensitivity (Hein et al., 2006; Rolke et al., 2008). It has been argued that attention facilitates the processing at parvocellular neurons which increases the spatial resolution but at the same time decreases temporal resolution (Yeshurun & Carrasco, 1998). In our Experiment 1, sensitivity was lower with tactile cues compared to visual ones. This difference may be due to tactile cues directing spatial attention while the visual cues did not have the same effect. Thus, we extended findings that are established for the visual and auditory modalities to the tactile modality on the human torso. Since brief tactile cues on the torso have been demonstrated to be effective in directing attentional resources to the area which is cued (Spence & Gallace, 2007), and attention is known to cause expansion of duration judgments (Seifried & Ulrich, 2011) and decrease in temporal sensitivity, our results corroborate the assumption that attention affects timing on a general, modality-independent level.

Unexpectedly, endogenous visual cues from the same side as the tactile stimulus did not affect tactile timing judgments on the torso in Experiment 1. This lack of effect might be because crossmodal spatial attention is most effective when cue and stimulus can be bound together spatially (Spence, 2010). That is, both cue and stimulus locations should be relatively similar for cueing to attract attention to the location of the stimulus. Our results can be interpreted such that, visually cueing from a screen 60 cm away cannot trigger attention to the human torso. Spatial thresholds between visual and tactile modalities in the scope of crossmodal attentional orienting should be further investigated to better understand the fundamental relationship between these modalities.

Experiment 2 showed that after dynamic tactile cues, subsequent tactile stimuli are felt to be longer than after static cues. Motion has been known to expand perceived time, particularly in visual perception (Kanai et al., 2006; Kaneko & Murakami, 2009) and because of its attention-capturing effects (Ono & Kitazawa, 2010; van Wassenhove et al., 2008). Our results extend this knowledge to the subjective timing judgments in presence of exogenous tactile cues. This result can be explained by the efficacy of dynamic tactile cues in capturing attention as shown by Ho and colleagues (2014). In that study, the authors demonstrated that dynamic cueing yielded significant performance advantages over static cueing, resulting in decreased response times to the subsequent target visual stimuli. We argue that subjective time judgments are also affected by the increased attentional capturing efficacy of dynamic cues on the human torso.

Unexpectedly, expansion of subjective timing was found in both congruent and incongruent spatial cue conditions in Experiment 2. Cueing on one side of the torso expanded the perceived time of tactile stimuli on both sides. This result can imply that the tactile cues oriented the spatial attention to the whole torso instead of to the right or the left side. Since the relative distance between two cue locations was only moderate, the cues might not have been sufficiently separated. Furthermore, as vibration has a dissipative characteristic, the stimuli can be perceived not only from the location of the actuator but also from a brief distance from its center. Hence the outer bounds of the area in which the cues were perceived became more adjacent to each other for both cued locations. However, it has been previously found that congruency affects spatial attention when the cue and target locations are front and back of the torso (Ho et al., 2014). More importantly, in Experiment 3 we used the front and back of the torso to present distinctly separate cue and target locations in the incongruent condition. There, we found that timing judgments were longer in the congruent condition, confirming an effect of spatial attention on timing judgments. Thus, congruency effects were only present if the locations were sufficiently separate. Overall, these results suggest that attentional orientation and the subsequent time perception on the human torso is not highly spatialized and, thus, tactile cues can be utilized to attract attention to a larger area of the torso. However, a separation between front and back of the torso was sufficient to attract attention explicitly to these specific areas.

Finally, in Experiment 4, we investigated if time expansion effects would vary with the cue-target intervals corresponding to the typical dynamics of spatial attentional processes. There was a significant interaction effect between cue-target interval and congruency: Post hoc tests showed that congruent as compared to incongruent cues lead to increased duration judgments in a 100-ms cue-target interval. Numerically (Fig. 10), such cue effects were smaller for larger intervals of 300 and 500 ms, and absent for the 5-ms interval. This is in line with spatial attention being maximal with a certain delay after a (congruent) cue (Chica et al., 2014; Posner & Cohen, 1984), and thus further corroborates that tactile cues can expand time perception mediated by spatial attention. At the same time, the data from Experiment 4 rule out a simple alternative explanation of our cue effects, namely that time expansion could be due to an integrated or summary temporal perception of cue and target. In this case we should have also seen time expansion in the congruent 5-ms delay condition, in that cue and target almost build a single unit due to their spatial alignment and small temporal separation.

One general explanation of attentional effects on subjective timing is related to scalar expectancy theory (Church, 2003; Gibbon, 1977). According to the pacemaker-accumulator model of the scalar expectancy theory, the internal clock system that is utilized in estimating the timing of events consists of a pacemaker, an accumulator, and a switch. The pacemaker emits pulses. These pulses are then accumulated in the accumulator where they are counted, which provides the basis for duration estimates. To measure time the switch opens to let pulses through to the accumulator, and the switch closes, stopping the accumulation process. Attention can affect timing as follows (Lejeune, 1998): Increased attention can result in opening the switch earlier or closing it later, which will both increase the periods in that the switch is open. Thus, this increase results in transmitting more pulses and increased duration judgments.

Exogenous cues have also been demonstrated to impair temporal resolution, which might contribute to the attention-related expansion of timing: Yeshurun and Levy (2003) investigated the two-flash fusion threshold with spatial cues. This threshold refers to the maximal latency between two visual flashes for them to be perceived as one flash (Tong & Ground, 1970). They found that the ability to detect separate flashes decreases with attentional cues. These results are related to an impairment in detecting the offset of the first flash after a cue. Hein and colleagues (2006) extended these findings by reporting the impairing of temporal order judgments by exogenous cues. Both results indicate a disruption in temporal mechanisms that fail to capture smaller time intervals in the focus of attention. This disruption can also explain expanded time judgments in the presence of exogenous cues: With worse resolution, it should be more difficult to capture the temporal offset of the judged stimuli. It has been shown that attention delays the perceived offset of a stimulus and this was related to the degradation in temporal resolution (Rolke et al., 2006). Our results align with this finding. Exogenous cueing on the human torso may have impaired the temporal resolution for tactile stimuli in the same location, the capture of offset was delayed, and thus the perceived duration was expanded.

Overall, we found that exogenous cues on the human torso expand the perceived timing of a tactile stimulus, and that dynamic cues are particularly effective in doing so. Furthermore, we showed that this effect varies with the cue-target interval. By extending the knowledge on audition and vision, we showed that attentional effects on time perception are present in the tactile modality on the human torso. Thus, the effect of attention on subjective timing can be considered supramodal, given it also generalizes to the tactile modality. Furthermore, in light of these results, attentional effects on the human torso can be considered as modulators of time perception. Thus, they could be utilized in applications (Botev et al., 2021) where expanding or contracting the subjective timing is crucial without populating the already crowded auditory and visual channels.

## Supplementary Information

Below is the link to the electronic supplementary material.

Supplementary file1 (DOCX 17 KB)

## Data Availability

Raw data are available from the public repository on the link: https://zenodo.org/records/14865278
