# Supplementary material for: Spatial attention modulates time perception on the human torso
Source: Atten Percept Psychophys. 2025 Feb 19;87(3):779–93. doi: 10.3758/s13414-025-03025-6 (PMC11965207; doi:10.3758/s13414-025-03025-6)
Supplement: Supplementary file 1 — Supplementary file1 (DOCX 17 KB) [file 13414_2025_3025_MOESM1_ESM.docx]

**Supplementary Table 1**

*Repeated Measures ANOVA Results of Just Noticeable Differences in Experiment 1*

|  | df | *F* | p | η^2^_p_ |
| --- | --- | --- | --- | --- |
| Cueing | (2, 50) | 1.295 | .283 | .04 |
|  |  |  |  |  |

**Supplementary Table 2**

*Bonferonni corrected post-hoc tests on JNDs in Experiment 1*

| Condition1 | *Condition 2* | *Mean Difference* | Standard Error | t | p_bonf_ |
| --- | --- | --- | --- | --- | --- |
| No Cue | Haptic Cue | -.002 | .003 | .496 | 1.000 |
| No Cue | Visual Cue | .004 | .003 | 1.078 | .859 |
| Haptic Cue | Visual Cue | .005 | .003 | 1.574 | .366 |

**Supplementary Table 1**

*Repeated Measures ANOVA Results of Just Noticeable Differences in Experiment 1*

|  | df | *F* | p | η^2^_p_ |
| --- | --- | --- | --- | --- |
| Congruency | (1, 23) | .910 | .350 | .0 |
| Movement | (1, 23) | .003 | .959 | .001 |

**Supplementary Table 3**

*Repeated Measures ANOVA Results of Just Noticeable Differences in Experiment 3*

|  | df | *F* | p | η^2^_p_ |
| --- | --- | --- | --- | --- |
| Congruency | (1, 23) | .910 | .350 | .038 |
| Movement | (1, 23) | .003 | .959 | .001 |
| Torso Side | (2, 46) | .142 | .868 | .006 |
| Congruency* Movement |  |  |  |  |
|  |  |  |  |  |
